# Dopamine Receptor D3 Induces Transient, mTORC1-Dependent Autophagy That Becomes Persistent, AMPK-Mediated, and Neuroprotective in Experimental Models of Huntington’s Disease

**DOI:** 10.3390/cells14090652

**Published:** 2025-04-29

**Authors:** Diego Luis-Ravelo, Felipe Fumagallo-Reading, Alejandro Febles-Casquero, Jonathan Lopez-Fernandez, Daniel J. Marcellino, Tomas Gonzalez-Hernandez

**Affiliations:** 1 Institute of Biomedical Technologies, University of La Laguna, 38200 Tenerife, Spain; dluisrav@ull.edu.es (D.L.-R.); fumaga11o.tf@gmail.com (F.F.-R.);; 2 Department of Basic Medical Sciences, Faculty of Medicine, University of La Laguna, 38200 Tenerife, Spain; 3 Department of Medical and Translational Biology, Umeå University, 901 87 Umeå, Sweden; daniel.marcellino@psy.gu.se

**Keywords:** dopamine receptors, Huntington’s disease, neuroprotection, ULK1, mTORC1, AMPK

## Abstract

Huntington disease’s (HD) is a neurodegenerative disorder caused by the expansion of a polyglutamine region (PolyQ) within the huntingtin protein (HTT). Mutated huntingtin (mHTT) is cytotoxic, particularly for striatal medium spiny neurons (MSNs), whose degeneration is the hallmark of HD. Autophagy inducers currently available promote the clearance of toxic proteins. However, due to their low selectivity and the possibility that prolonged autophagy hampers essential processes in unaffected cells, researchers have questioned their benefits in neurodegenerative diseases. Since MSNs express dopamine receptors D2 (DRD2) and D3 (DRD3) and DRD2/DRD3 agonists may activate autophagy, here, we explored how healthy and mHTT-challenged cells respond to prolonged DRD2/DRD3 agonist treatment. Autophagy activation and its effects on mHTT/polyQ clearance were studied in R6/1 mice (a genetic model of HD), their wild-type littermates, and *DRD2*- and *DRD3*-HEK cells expressing a pathogenic (Q74) and a non-pathogenic (Q23) polyQ fragment of mHTT treated with the DRD2/DRD3 agonist pramipexole. Two forms of DRD3-mediated autophagy were found: a transient mTORC1-dependent in WT mice and *Q23-DRD3*-HEK cells and a persistent AMPK-ULK1-activated in R6/1 mice and *Q74-DRD3*-HEK cells. This also promoted a robust clearance of soluble mHTT/polyQ and neuroprotection in striatal neurons and *DRD3*-HEK cells. The findings indicate that DRD3-induced autophagy may be a safe, disease-modifying intervention in HD patients.

## 1. Introduction

Neurodegenerative diseases are a heterogeneous group of neurological conditions characterized by the formation and accumulation of misfolded proteins that cause neuronal dysfunction and degeneration. There are currently no disease-modifying therapies for neurodegenerative diseases. Research is focused on strategies that block synthesis, prevent misfolding, or promote the degradation of aberrant protein conformations [1,2]. Autophagy is a degradative process essential for neurons, as they must deal with misfolded proteins throughout their lifetime without the aid of cell division. Macroautophagy is a form of autophagy activated by the mechanistic target of rapamycin kinase (mTOR)-dependent and mTOR-independent signaling in which aggregate-prone proteins are engulfed in double-membraned vesicles and degraded by lysosomes [3,4]. Experimental studies demonstrate that macroautophagy (hereafter referred to as autophagy) disruption is a central factor in the pathogenesis of neurodegenerative diseases [5,6] and that its induction protects against degeneration and improves motor and cognitive phenotypes in various animal models of neurodegenerative disease [7]. However, data also suggest that prolonged autophagy activation poses risks. For example, autophagy may deplete proteins involved in cell homeostasis, interfere with protein synthesis, and interact with the apoptotic machinery, affecting cell viability and leading to cell death [8,9,10]. Therefore, as the available autophagy inducers act non-selectively on both degeneration-affected and non-affected neurons, the beneficial effects of autophagy on degenerating cells might be overshadowed by their deleterious effects on non-degenerating cells.

Huntington’s disease (HD) is an autosomal-dominant, neurodegenerative disorder characterized by psychiatric, cognitive, and motor symptoms that appear at 35–45 years of age and lead to death in 15–20 years [11]. HD is caused by an expansion of the trinucleotide CAG repeat (>35 repeats) in the gene that encodes huntingtin (HTT), resulting in an abnormally long polyglutamine (polyQ) tract at the *N*-terminal region of the protein (polyQ-HTT). Proteolytic fragments of polyQ-HTT are prone to misfold and aggregate into soluble oligomers and insoluble inclusion bodies [12]. Soluble mutant huntingtin (mHTT) oligomers can interact with key proteins, interfering with their functions and leading to cell death [13,14]. Medium spiny neurons (MSNs) of the striatum are particularly vulnerable in HD [15,16]. Their loss leads to motor symptoms and striatal atrophy, the two hallmarks of the disease [16,17].

Interestingly, striatal MSNs express dopamine receptors D2 and D3 (DRD2, DRD3) [18,19], and recent studies indicate that DRD2/DRD3 agonists can promote autophagy through a DRD3- and mTORC1-mediated mechanism [20]. So, by acting selectively on DRD3-expressing neurons, we might avoid the adverse effects of prolonged autophagy on other neuronal populations. However, as HTT regulates mTORC1, and mTORC1 is constitutively overactivated in mHTT-expressing cells [21], the response of MSNs to DRD2/DRD3 agonists in HD could differ from that under normal conditions. Furthermore, since not all DRD3-expressing neurons are equally affected by degeneration at the same stage of HD, the final effect of prolonged autophagy may vary from one neuron to another. This study aimed to investigate whether DRD3-induced autophagy is activated and maintained in experimental models of HD and determine its effects on healthy and polyQ-HTT-challenged cells. Using cell and animal models of HD, we found two forms of DRD3-induced autophagy: a transient form in healthy cells activated through mTOR inhibition and a persistent form in polyQ-HTT-challenged cells that was prolonged through AMPK activation. Considering the absence of deleterious effects on protein synthesis [20], this autophagy form promoted the efficient clearance of mHTT without affecting cell homeostasis.

## 2. Materials and Methods

### 2.1. Mice

The experiments were carried out on the transgenic mouse strain R6/1 (B6.Cg-Tg(HDexon1)61Gpb/J Jackson Laboratory, Bar Harbor, ME, USA), dopamine receptor D3 knockout mice (*drd3*KO; B6.129S4-Drd3^tm1Dac^/J, Jackson Laboratory), and their wild-type (WT) littermates. Experimental procedures were approved by the Ethical Committee of the University of La Laguna (reference: CEIBA2019-0352) in accordance with the ARRIVE guidelines and the European Communities Council Directive of 22 September 2010 (2010/63/EU) regarding the care and use of animals for scientific purposes. Mice were housed in groups of 4–5 animals per cage with ad libitum access to feed and water on a 12 h dark/light cycle at a constant temperature (21–22 °C). R6/1 mice were bred in a C57BL/6 background by mating hemizygous males (6–10 weeks) with 2–3 females per cage. Genotype was determined by PCR [22]. The transgene contains an N-terminal fragment of human *HTT* encompassing the first exon, with around 116–120 CAG repeats. Mice inheriting the mutated allele develop most HD symptoms [23], with reduced motor activity from 6–7 weeks onward [24]. Hemizygous R6/1 transgenic male mice and age- and body weight-matched wild-type littermates were randomly assigned to experimental groups and treated with pramipexole. Pramipexole (PPX) is a DRD2/DRD3 agonist with a preference for DRD3 (DRD2 Ki 6.9 nM, DRD3 Ki 0.9 nM), which is extensively used and generally well-tolerated in controlling motor symptoms of Parkinson’s disease and restless leg syndrome [25,26]. Mice received a daily dose of 0.15 mg/kg of pramipexole dihydrochloride (Sigma-Aldrich, St. Louis, MO, USA; #A1237) or its vehicle (100 µL 0.9% sterile saline i.p.) for 6 days (11-week-old mice) or 28 days (starting at 8 weeks old) and were sacrificed on day seven or twenty-nine, respectively. This PPX dose is effective as a DRD2/DRD3 agonist in rodents [27,28]. It is equivalent to a human dose of 0.72 mg/d [29] within the therapeutic range used in Parkinson’s disease patients. An additional group of R6/1 mice was treated with the selective DRD3 antagonist NGB2904 (DRD2 Ki 217 nM, DRD3 Ki 1.4 nM; 0.5 mg/kg, i.p.; Tocris, Bristol, UK; #2635) or vehicle (2.5% *w*/*v* 2-hydroxypropyl-β-cyclodextrin; Sigma-Aldrich; #H107) 30 min before PPX. Mice were weighed weekly and bedding was replaced twice a week. Each experimental group consisted of at least five mice.

### 2.2. Cell Culture and Stable DRD2/DRD3 Transfection

Cell culture studies were performed in *DRD2*- and *DRD3*-transfected HEK 293 cells. We used HEK293 cells because they endogenously express most proteins involved in G protein-coupled receptor signaling but not dopamine receptors [30,31]. This prevents the impact of other dopamine receptors present in other neuronal cell lines. HEK293 cells were obtained from the European Collection of Cell Cultures (ECACC, Salisbury, UK), cultured in Dulbecco’s Modified Eagle’s Medium (DMEM; Biowest, Nuaillé, France; #L104) supplemented with 10% fetal bovine serum (FBS; Biowest; #S1810-500) and 1% penicillin–streptomycin (Biowest; #L0018), and maintained in a humidified incubator set at 37 °C and 5% CO_2_. Semiconfluent cells were transfected with pcDNA3.1+/Hygro-GFP-*DRD2* or pCEP4-EGFP-*DRD3* (a gift from Dr. Jean-Michel Arrang, Addgene, Watertown, MA, USA; #24098 and #24099; [32]) using Lipofectamine 2000 (Thermo Fisher Scientific, Waltham, MA, USA; #11668-027) at a ratio of 2:1. Stable cell lines expressing GFP-DRD2 or EGFP-DRD3 were obtained by growth in selective medium containing 400 μg/mL hygromycin B (Thermo Fisher Scientific; #1068-7010) over 8–10 days. Individual clones were expanded in 96-well plates and examined for expression by Western blot and fluorescence for GFP (Figure S1A,B). Positive clones were used for subsequent experiments. Cell treatments are stated throughout the text and figure legends.

### 2.3. Transient Q23/Q74 Transfection

For the transient expression of polyglutamine tracts, HEK-, *DRD2*-HEK, and *DRD3*-HEK cells were transfected with plasmid encompassing a fragment of huntingtin exon 1 containing either 23 or 74 CAG repeats cloned downstream from the tag (*pEGFP-Q23* or pHM6 (*HA*)*-Q74*; gifts from Dr. David Rubinsztein, Addgene; #40261 and #40264, respectively [33]; Figure S1C). Transfections were performed in 10 cm cell culture dishes using DNA–PEI (Polysciences Inc, Warrington, PA, USA; #23966) at a ratio of 1:3. Since soluble polyQ oligomers reach their highest levels a few hours after transfection and drop as polyQ aggregates arise [13,14] (see also Figure S1D), treatment was initiated six hours post-transfection. Cells were split into 12-well plates and treated with PPX at the indicated doses and times. An additional set of *DRD3*-HEK cells was treated with another DRD2/DRD3 agonist, 7OH-DPAT (DRD2 Ki 61 nM, DRD3 Ki 0.78 nM; Figure S2A).

### 2.4. Western Blot

Mouse striata were dissected on ice from freshly obtained brains using a brain blocker. Samples were subjected to sonication and then lysed in M-PER buffer (Thermo Fisher Scientific; #78501) supplemented with a protease and phosphatase inhibitor (Sigma-Aldrich; #4906845001). Lysates were centrifuged at 9000× *g* and protein-containing supernatants were collected. HEK293 cells were harvested in ice-cold, phosphate-buffered saline (PBS; 137 mM NaCl, 2.7 mM KCl, 10 mM Na_2_HPO_4_, 1.8 mM KH_2_PO_4_; pH 7.4). Cell suspensions were centrifuged for 5 min at 1000× *g*; the pellets were washed twice in PBS and then resuspended in M-PER buffer. After sonication (3 bursts of 5 s on ice), the lysates were centrifuged at 17,000× *g* for 5 min and the supernatants were collected. Proteins were quantified using the bicinchoninic acid (Sigma-Aldrich; #B9643) method, with bovine serum albumin (BSA; Sigma-Aldrich; #1076192) as the standard. Protein samples were diluted in Laemmli’s loading buffer (62.5 mM Tris-HCl, 20% glycerol, 2% sodium dodecyl sulfate, 1.7% β-mercaptoethanol, 0.05% bromophenol blue; pH 6.8), denatured (95 °C, 5 min), separated by electrophoresis in 10–12% SDS-polyacrylamide gel (15% in the case of LC3 Western blot analysis), and transferred to nitrocellulose (Schleicher & Schuell, Dassel, Germany; #1620115) or PVDF (Bio-Rad, Hercules, CA, USA; #162-0175) membranes in the case of LC3 Western blot analysis. Blots were blocked for 1 h at room temperature (RT) with 5% non-fat dry milk (5% BSA for blots detecting phosphorylated forms) in TBST (250 mM NaCl, 50 mM Tris, pH 7.4, 0.05% Tween20) and incubated overnight at 4 °C in blocking solution (0.5% non-fat dry milk/BSA) with one of the following antibodies: mouse monoclonal anti-polyQ (Merck-Millipore, Burlington, MA, USA; #MAB1574, 1C2; 1:3000), rabbit polyclonal anti-DARPP-32 (Merck-Millipore; #AB10518; 1:40,000), rat monoclonal anti-HA (Sigma-Aldrich; #11867423001; 1:2000), mouse monoclonal anti-GFP (Roche, Basel, Swiss; #11814460001; 1:4000), rabbit polyclonal anti-LC3 (Sigma-Aldrich; #L7543; 1:15,000), guinea pig polyclonal anti-SQSTM1/p62 protein (Progen, Heidelberg, Germany; #GP62-C; 1:1000), rabbit polyclonal anti-TOLLIP (EMD-Millipore, Burlington, MA, USA; #ABF296; 1:2000), rabbit polyclonal anti-MAPK1/ERK2-MAPK3/ERK1 (R&D Systems, Minneapolis, MN, USA; #AF1576; 1:7000), rabbit polyclonal anti-phosphoThr202/Tyr204-MAPK1/ERK2-MAPK3/ERK1 (Cell Signaling Technology, Danvers, MA, USA; #9101; 1:5000), rabbit monoclonal anti-mTOR (Cell Signaling Technology; #2983; 1:4000), rabbit polyclonal anti-phosphoSer2448-mTOR (Merk-Millipore; #09213; 1:2000), rabbit polyclonal anti-PRKAA/AMPKα (Cell Signaling Technology; #2532; 1:2000), rabbit monoclonal anti-phosphoThr172-PRKAA/AMPKα (Cell Signaling Technology; #2535; 1: 2000), rabbit polyclonal anti-RPS6KB1/p70S6K (Cell Signaling Technology; #9202; 1:2000), mouse monoclonal anti-phosphoThr389-RPS6KB1/p70S6K (Cell Signaling Technology; #9206; 1:2000), rabbit monoclonal anti-RPS6KA/p90S6K (Cell Signaling Technology; #9355; 1:2000), rabbit monoclonal anti-phosphoSer380-RPS6KA/p90S6K (Cell Signaling Technology; #11989; 1:2000), rabbit monoclonal anti-rpS6 (Cell Signaling Technology; #2217; 1:3000), rabbit polyclonal anti-phosphoSer235/236-rpS6 (Cell Signaling Technology; #2211; 1:5000), rabbit monoclonal anti-ULK1 (Cell Signaling Technology; #8054; 1:2500), rabbit polyclonal anti-phosphoSer555-ULK1 (Cell Signaling Technology; #5869; 1:2500), rabbit polyclonal anti-phosphoSer757-ULK1 (Cell Signaling Technology; #14202; 1:2500), mouse anti-ACTB/β-Actin (Sigma-Aldrich; #A5441; 1:15,000), and anti-TUBA4A/α-Tubulin (Sigma-Aldrich; #T6074; 1:30,000). After several rinses in TBST-5% milk, the membranes were incubated for 1 h in 1:50,000 horseradish peroxidase-conjugated anti-mouse (Jackson-ImmunoResearch Laboratories, West Grove, PA, USA; #115-035-146), anti-guinea pig (Jackson-ImmunoResearch Laboratories; #106-035-003), anti-rabbit (Jackson-ImmunoResearch Laboratories; #111-035-144), or anti-rat (Jackson-ImmunoResearch Laboratories; #112-035-062) IgGs. Immunoreactive bands were visualized using enhanced chemiluminescence (Clarity Western Substrate; Bio-Rad; #170-5061) and a Chemi-Doc gel documentation system (Bio-Rad; #20089). Different protein quantities, antibody dilutions, and exposure times were tested to determine the optimal working range for each antibody. The labeling densities were compared using densitometry software (Image Lab 5.2, Bio-Rad) and β-actin or α-tubulin as housekeeping proteins, depending on the molecular weight of the protein being tested. A rectangle of uniform size and shape was placed over each band, and the density values were calculated by subtracting the background at approximately 2 mm above each band. Data are presented as percentages of their respective controls (100%).

### 2.5. Immunofluorescence

Mice were deeply anesthetized with an overdose of sodium pentobarbital and transcardially perfused with heparinized ice-cold 0.9% saline (20 mL) followed by 4% paraformaldehyde (Sigma-Aldrich) in PBS (50 mL). The brains were removed and maintained overnight in the same fixative at 4 °C. They were then cryoprotected in a 30% (*w*/*v*) sucrose–PBS solution and stored at –80 °C until processing. Coronal sections (25 µm) were obtained with a freezing microtome (Thermo Fisher Scientific), collected in 6–8 parallel series, and processed for immunofluorescence. Floating sections were washed three times in PBS and incubated for 60 min at room temperature (RT) in 4% normal donkey serum (NDS; Jackson ImmunoResearch; #017-000-121) in PBS containing 0.05% Triton X-100 (TX-100; Sigma-Aldrich). They were then incubated overnight in PBS containing 4% NDS and guinea pig anti-p62 antibody (1:500) or mouse anti-huntingtin monoclonal antibody (Merck-Millipore; Clone EM48; #MAB5374; 1:1000) in combination with rabbit anti-DARPP-32 antibody (1:2000). After several rinses, sections were incubated for 90 min with either biotinylated donkey anti-guinea pig antisera (Jackson ImmunoResearch; 1:200) followed by 1 h incubation with Extravidin-Cy2 (Jackson ImmunoResearch; #715-225-150; 1:1000) or Alexa Fluor 546-conjugated donkey anti-mouse (#A10036) and Alexa Fluor 488-conjugated (#A-21206) donkey anti-rabbit antisera (Thermo Fisher Scientific; 1:200) in 1:200 NDS in PBS. Slices were mounted using Vectashield Mounting Medium with DAPI (SouthernBiotech, Birmingham, AL, USA) and analyzed under a confocal laser scanning microscopy system (Leica TCS SP8, Wetzlar, Germany).

Untransfected and *Q23*- and *Q74*-transfected *EGFP-DRD3*-HEK cells were fixed in 4% paraformaldehyde in PBS for 20 min at RT. After several rinses in PBS containing calcium and magnesium (PBS Ca^2+/^Mg^2+^), the coverslips were permeabilized with 0.1% Triton X-100 in PBS Ca^2+/^Mg^2^ for 10 min at RT, washed, and blocked with 1% FBS. The cells were then incubated overnight at 4 °C with rat anti-HA (1:200) and mouse anti-phospho-Histone H2AX Ser139 (γH2AX, Merck-Millipore; #05-636-I; 1:350) in blocking solution. Afterward, they were rinsed in PBS and incubated with Alexa Fluor 647-conjugated goat anti-rat (Abcam, Cambridge, UK; #ab150159; 1:500) and biotinylated donkey anti-mouse (Jackson Immunoresearch; #115-065-003; 1:200) antisera, followed by Cy3-conjugated streptavidin (Jackson Immunorsearch; #016-160-084; 1:1000). After further washing, the coverslips were mounted with Vectashield Mounting Medium with DAPI. The specificity of γH2AX immunolabeling was confirmed in *DRD3*-HEK cells treated overnight with the DNA damage inducer doxorubicin (Sigma-Aldrich; #D29775000; 1 µM) and replacing the primary antibody with non-immune IgG in negative controls (Figure S1E). Samples were examined under confocal laser scanning microscopy.

### 2.6. Quantitative and Densitometric Analysis in Immunofluorescence Material

The number and size of intranuclear inclusions and the intensity of DARPP-32 labeling in mouse striata, as well as the number of pyknotic nuclei and intensity of polyQ and γH2AX labeling in HEK cells, were quantified by using the ImageJ standard program (RRID: SCR_003070). Four sections, 100–125 µm apart from each other, were randomly selected at the rostrocaudal level, between Bregma 0.98 mm and 0.50 mm [34], in five mice per experimental group. For the analysis of intranuclear inclusions, images were acquired at 60X magnification (1024 × 1024 pixels) in Z-stack mode, with a total thickness of 9 μm and 5 z-steps. Six 150 µm × 150 µm striatal fields were randomly selected from each animal. At least 140 cells were analyzed per animal. The inclusion size was measured in single cells at the section where the nucleus had the largest diameter (Figure S1F). The size of intranuclear inclusions is expressed as a percentage of the average inclusion size in vehicle-treated R6/1 mice (100%). For the analysis of DARPP-32 labeling intensity, images were acquired at 10× magnification, and 8 fields (5 in the dorsal striatum and 3 in the ventral striatum; 400 µm × 400 µm) were randomly selected from each animal. Twelve 220 µm × 220 µm fields of 80% confluent cells were randomly selected from three different coverslips of each experimental condition for counting pyknotic nuclei in cell cultures. For the densitometric analysis of polyQ and γH2AX labeling intensity, images were acquired at 60× magnification (1024 × 1024 pixels). Twelve 50 µm × 50 µm fields were randomly selected from three different coverslips of each experimental condition. Square areas of 5 µm × 5 µm of at least 10 randomly selected cells per field were analyzed. The intensity of immunofluorescent labeling in striatal sections and cell samples is expressed in arbitrary units (range: 0–256). To prevent differences due to variations in protocol conditions during sample processing and analysis, all sections and cell samples were processed simultaneously using the same protocol and reagents, and all microscopic and computer parameters were maintained at a constant level throughout this study.

### 2.7. Motor Behavior

The open field test was used to evaluate motor activity. Tests were performed during the fourth week of treatment and 20–22 h after PPX administration. ActiMot2 (TSE Systems, Berlin, Germany) activity monitors were used to quantify the distance traveled (in cm) and speed (in cm/s). The open field boxes (515 × 515 × 400 mm^3^) were in a sound-protected, dimly illuminated room. Spontaneous activity was registered for 60 min, and all the experiments were performed from 8:30 AM to 2:30 PM. The XY infrared beams were spaced 27 mm apart and 40 mm above the floor. The distance traveled was measured by the sequential beam breaking of infrared light within the horizontal plane defined by the X- and Y-axes. The average speed was calculated as the total horizontal distance divided by the total time in motion.

### 2.8. Statistics

Data were plotted using GraphPad Prism 5 software (San Diego, CA, USA) and presented as the mean ± SEM. The statistical tests are described in the figure legends throughout the text. Unless specified in the figure legend, the unpaired *t*-test or Mann–Whitney U test was performed for parametric or non-parametric analysis, respectively. ANOVA followed by the Tukey/Holm–Sidak multiple comparison test was performed for two experimental groups. Kruskal–Wallis, followed by Dunn’s multiple comparison test, was performed when comparing more than two experimental groups. Data from all experiments performed were included. A *p*-value of less than 0.05 was considered statistically significant. 

## 3. Results

### 3.1. DRD3-Induced Autophagy Is Transient in WT Mice and Q23-Expressing Cells and Persistent in R6/1 Mice and Q74-Expressing Cells

**Figure 1:**
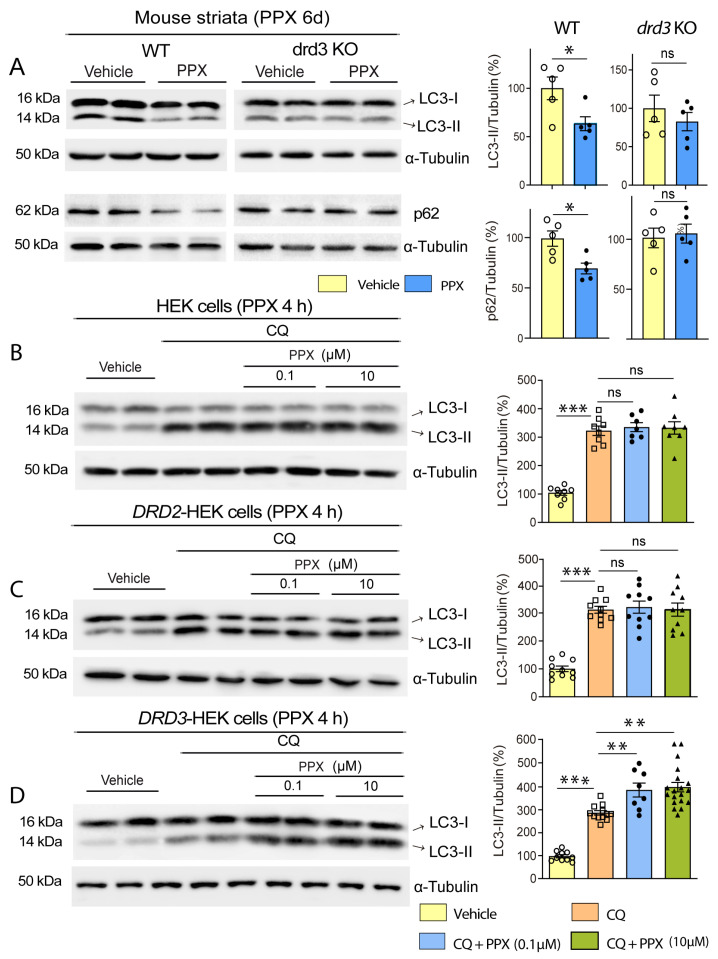
PPX induces autophagy through a DRD3-dependent mechanism in mouse striata and HEK cells. (**A**) Western blot and densitometric analysis for LC3 and p62 in WT and *drd3*KO mice treated with 0.15 mg/kg/d for six days (*n* = 5). PPX promotes a decline in LC3-II and p62 in WT but not in *drd3*KO mice. (**B**–**D**) Autophagic flux in HEK (*n* = 7–8; (**B**)), *DRD2*-HEK (*n* = 10; (**C**)), and *DRD3*-HEK (*n* = 8–19; (**D**)) cells treated with the autophagosome–lysosome fusion blocker chloroquine (CQ, 20 µM, 1 h; lanes 3 and 4) and 0.1 µM PPX (lanes 5 and 6) or 10 µM PPX (lanes 7 and 8) for 4 h. CQ was added after PPX. Western blot for LC3 revealed that autophagic flux (LC3-II levels in CQ + PPX vs. CQ) was increased after PPX treatment in *DRD3*-HEK cells (**D**) but not in HEK (**B**) or *DRD2*-HEK cells (**C**). Statistical analyses were performed using the unpaired *t*-test for mice and Mann–Whitney test for cells. *n* = number of mice per experimental group or number of experimental repeats in cells; ns = non-significant; * *p* < 0.05; ** *p* < 0.01; *** *p* < 0.001.

Considering that striatal MSNs express both DRD2 and DRD3 [18,19] and that the high structural homology between DRD2 and DRD3 [35] has hindered the design of selective DRD2 or DRD3 agonists, in the first set of experiments, WT and *drd3*KO mice were treated with PPX (0.15 mg/kg i.p.) for 6 days to confirm previous data indicating that autophagy induced by DRD2/DRD3 agonists is mediated by DRD3 [20]. The autophagy markers microtubule-associated protein 1 light chain 3 (MAP1LC3/LC3) and sequestosome 1 (SQSTM1/p62) were analyzed in striatal extracts using Western blot. LC3 exhibited two distinct bands: LC3-I, corresponding to the cytosolic fraction, and LC3-II, corresponding to the phosphatidylethanolamine-conjugated fraction. This form is recruited to autophagosomal membranes during autophagosome formation and is considered an index of the number of autophagosomes [36]. LC3-II levels may be increased due to a boost in autophagosome biogenesis or a decline in their degradation or decreased as a result of a reduction in autophagosome biogenesis or an increase in autophagy flux [37,38]. Therefore, changes (either increases or decreases) in LC3-II levels indicate the modulation of autophagy. However, this is insufficient to confirm whether autophagy is induced or inhibited, and the parallel analysis of other markers is required [38]. p62 is an autophagy receptor widely used as an autophagy marker degraded with LC3-II and its ubiquitinated cargos by lysosomes [38,39]. As shown in Figure 1A, after PPX treatment, LC3-II and p62 were reduced in the striatum of WT but not in *drd3*KO mice, suggesting that PPX increases autophagy flux through DRD3.

Autophagy flux was also studied in *DRD3*-, *DRD2*-, and non-transfected HEK cells treated with PPX (0.01 µM–10 μM) for 4 h, using chloroquine (CQ, 20 μM, 1 h) as an autophagosome–lysosome fusion blocker. As expected, CQ substantially increased LC3-II levels in transfected and non-transfected HEK cells (Figure 1B–D, compare lanes 1 and 2 with lanes 3 and 4). However, the addition of PPX (CQ + PPX) only induced a further increase in LC3-II in *DRD3*-HEK cells. This effect was observed at PPX concentrations ≥ 0.1 µM. (Figure 1B–D; compare lanes 3 and 4 with lanes 5 to 8). In summary, these data indicate that PPX-induced autophagy is DRD3-dependent.

**Figure 2:**
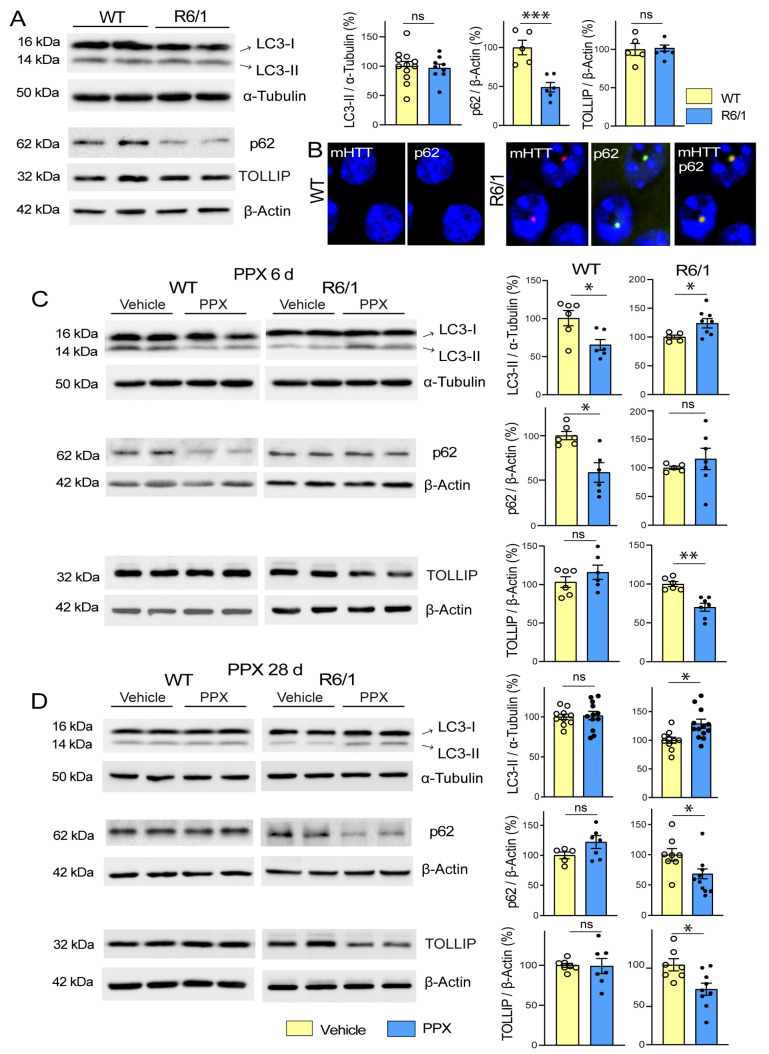
PPX induces transient autophagy in the striatum of WT mice and persistent autophagy in R6/1 mice. (**A**) Basal expression of autophagy markers in the striatum of 12-week-old WT and R6/1 mice. Western blot showed no differences in LC3-II (*n* = 9–12) or TOLLIP (*n* = 5), while p62 levels were significantly reduced in R6/1 mice (*n* = 5–6). (**B**) Immunofluorescence for mHTT (red) and p62 (green) in striatal neurons of WT and R6/1 mice showing p62 accumulation in intranuclear inclusions. Nuclei were stained with DAPI (blue). Bar: 10 µm. (**C**,**D**) Comparative analysis of autophagy markers in the striatum of WT and R6/1 mice after 6 days (**C**) and 28 days (**D**) of PPX treatment (0.15 mg/Kg/d). In WT mice, LC3-II and p62 expression was reduced after 6 days of treatment (*n* = 6), but no changes were detected after 28 days of treatment (*n* = 5–11). In R6/1 mice, LC3-II was increased and TOLLIP reduced after 6 days (*n* = 5–7) and 28 days (*n* = 6–13) of treatment, when p62 was also reduced. Statistical analyses were performed using the unpaired *t*-test. *n* = number of mice per experimental group; ns = non-significant; * *p* < 0.05; ** *p* < 0.01; *** *p* < 0.001.

Thereafter, DRD3-induced autophagy was evaluated in the striatum of WT and R6/1 mice. The autophagy receptor TOLLIP was also assessed in these experiments because it has a higher affinity for polyQ tracts than p62 [40,41]. Under basal conditions, the autophagy markers LC3-II and TOLLIP showed no differences between both strains, but p62 was significantly reduced in R6/1 mice (Figure 2A). It should be noted that p62 is sequestered into intranuclear inclusions in the brain of HD patients and animal models (Refs. [42,43]; see also Figure 2B), which may underlie the lowering of its soluble fraction in Western blot. WT and R6/1 mice were then treated with PPX for 6 days and 28 days. As described in experiments in WT and *drd3*KO mice, after 6 days of treatment, LC3-II and p62 levels were decreased in WT mice, but no changes were detected in TOLLIP (Figure 2C, left). In R6/1 mice, in contrast, LC3-II levels were increased, no significant changes were detected in p62, and TOLLIP was significantly reduced (Figure 2C, right). When PPX treatment was prolonged for 28 days, LC3-II and p62 returned to basal levels in WT mice (Figure 2D, left). However, the increase in LC3-II and the decrease in TOLLIP were maintained, and p62 was also reduced in R6/1 mice (Figure 2D, right). The results indicate that autophagy is initially induced in the striatum of both wild-type (WT) and R6/1 mice; however, when the treatment is prolonged, it remains activated only in R6/1 mice.

**Figure 3:**
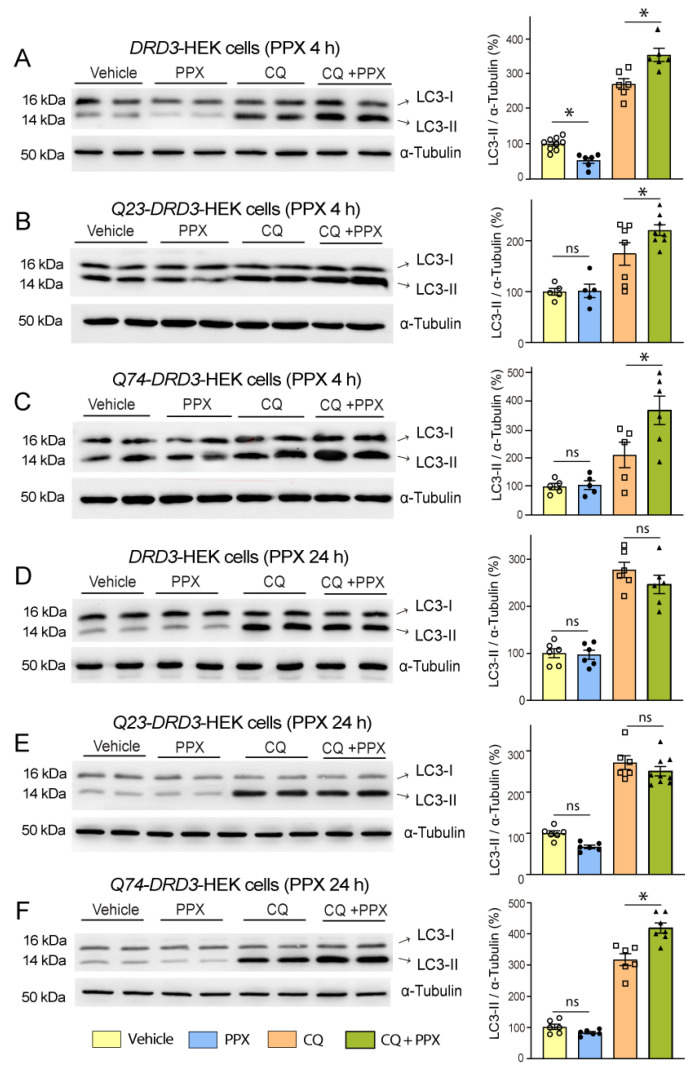
DRD3-induced autophagy is prolonged in cells expressing a pathogenic form of polyQ-HTT. The effects of PPX (0.1 µM) on LC3-II levels were analyzed in the absence (lanes 3 and 4) and presence (lanes 7 and 8) of the autophagosome–lysosome fusion blocker chloroquine (CQ, 20 µM, 1 h; lanes 5 and 6). (**A**–**C**) After 4 h of PPX treatment (*n* = 5–9), the autophagic flux (LC3-II levels in CQ + PPX vs. CQ) was increased in *DRD3*- (**A**), *Q23-DRD3*- (**B**), and *Q74-DRD3*-HEK (**C**) cells. (**D**–**F**) After 24 h of PPX treatment (*n* = 6), the autophagic flux remained increased in *Q74-DRD3*-HEK cells (**D**) but not in *DRD3*-HEK (**E**) or *Q23-DRD3*-HEK cells (**F**). Statistical analyses were performed using the Mann–Whitney test. *n* = number of experimental repeats; ns = non-significant; * *p* < 0.05.

The relationship between HTT-polyQ expression and autophagy persistence was also studied in HEK cells. Non-transfected *DRD3*-HEK cells and *DRD3*-HEK cells transiently transfected with a non-pathogenic (Q23) or pathogenic (Q74) form of polyQ were treated with 0.1 µM PPX for 4 h and 24 h. After 4 h of PPX treatment, autophagy flux was increased in the three cell phenotypes (Figure 3A–C; compare lanes 5 and 6 with lanes 7 and 8). However, when PPX treatment was prolonged for 24 h, autophagic flux was maintained in *Q74-DRD3*-HEK cells but not in non-transfected *DRD3*-HEK cells and *Q23-DRD3*-HEK cells (Figure 3D–F; compare lanes 5 and 6 with lanes 7 and 8). Taken together, the results reveal two temporal patterns of DRD3-mediated autophagy: transient in WT mice and *DRD3*- and *Q23-DRD3*-HEK cells and persistent in R6/1 mice and *Q74-DRD3*- HEK cells.

### 3.2. DRD3-Induced Autophagy Promotes mHTT Clearance and Neuroprotection in the Striatum of R6/1 Mice and Q74-DRD3-HEK Cells

**Figure 4:**
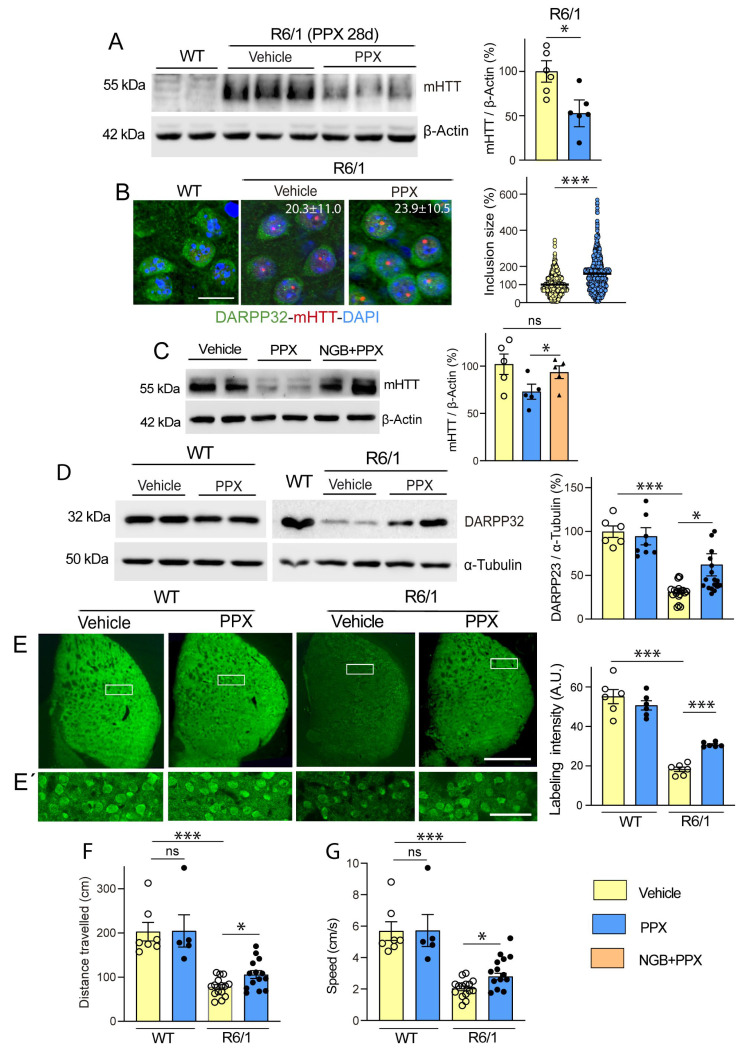
PPX promotes autophagic clearance of mHTT and protects striatal neurons against its cytotoxic effect. (**A**) Western blot for mHTT in WT mice (lanes 1 and 2) and R6/1 mice treated with vehicle (lanes 3–5) and PPX (lanes 6–8) for 28 days (*n* = 6). mHTT was not detected in striata of WT mice. The densitometric analysis showed a decrease in soluble mHTT in the striatum of R6/1 mice treated with PPX (unpaired *t*-test). (**B**) Immunofluorescence for DARPP-32 (green) and mHTT (red) and quantitative analysis of the size and number of intranuclear inclusions in striatal cells of R6/1 mice (*n* = 5). Nuclei were stained with DAPI (blue). The average size of inclusions in vehicle-treated R6/1 mice was taken as the reference value (100%). Numbers in the top right corner of the microphotographs indicate the number of intranuclear inclusions per field in each experimental group. Quantitative analysis revealed an increase in size (Mann–Whitney U test), without significant changes in the number of intranuclear inclusions (unpaired *t*-test) after PPX treatment. Bar: 10 µm. (**C**) Western blot and densitometric analysis of soluble mHTT in the striatum of R6/1 mice treated with PPX (lanes 3 and 4) or the selective DRD3 antagonist NGB2904 and PPX (NGB + PPX, lanes 5 and 6). The blockade of DRD3 prevented the decline of soluble mHTT induced by PPX (*n* = 5; Kruskal–Wallis test followed by Dunn’s multiple comparison test). (**D**,**E**,**E’**) Western blot (**D**) and immunofluorescence (**E**,**E’**) for DARPP-32 in the striatum of WT and R6/1 mice treated with PPX. (**E’**) corresponds to boxed areas in (**E**). Densitometric analysis showed that DARPP-32 expression was recovered after 28 days of PPX treatment in R6/1 mice. (*n* = 12–15 for Western blot; *n* = 6 for immunofluorescence; Brown–Forsythe ANOVA followed by Tamhane’s T2 multiple comparison test). A.U., arbitrary units. Bar: in (**E**): 1 mm; in (**E’**): 50 µm. (**F**,**G**) Analysis of locomotor activity in terms of total distance traveled (**F**) and speed (**G**). PPX improved both parameters of locomotor activity in R6/1 mice without affecting WT mice (*n* = 12–15 from three different experiments; Kruskal–Wallis followed by Dunn’s multiple comparison test). *n* = number of mice per experimental group; ns = non-significant; * *p* < 0.05; *** *p* < 0.001.

Western blot and immunofluorescence analyses using 1C2 and EM48 antibodies, respectively, demonstrated the robust expression of the soluble fraction of mHTT (Figure 4A, lanes 3–5) and intranuclear inclusions (Figure 4B) in the striatum of R6/1 mice. After 28 days of treatment with PPX, striatal levels of soluble mHTT significantly declined (~50%; Figure 4A, compare lanes 3–5 with lanes 6–8). Confirming that this effect is DRD3-mediated, mHTT lowering was prevented by treatment with the selective DRD3 antagonist NGB 2904 before PPX (Figure 4C). The quantitative analysis of intranuclear inclusions in striatal sections revealed that the decrease in soluble mHTT was paralleled by an increase in their size, without significant changes in their number (Figure 4B). On the other hand, the expression of dopamine- and cAMP-regulated phosphoprotein (DARPP-32), a marker of striatal medium spiny neurons [44] that is significantly reduced in R6/1 mice, was also partially restored by PPX, as shown by Western blot (Figure 4D) and immunohistochemistry (Figure 4E). Consistent with these findings, the analysis of motor activity in the open field test revealed a significant increase in the distance traveled and speed of R6/1 mice after PPX treatment (Figure 4F,G). PPX did not affect DARPP-32 expression or motor activity in WT mice.

**Figure 5:**
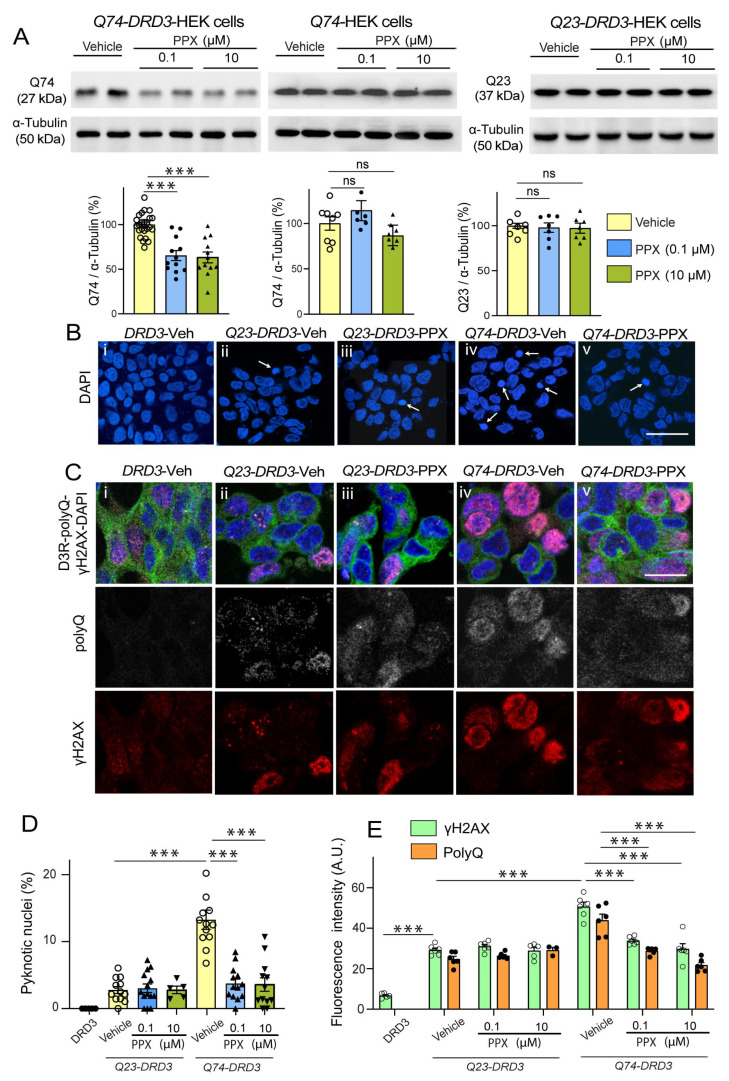
PPX promotes Q74 clearance and protects *DRD3*-HEK cells against its genotoxic effect. (**A**) Western blot for polyQ in *Q74-DRD3*- (*n* = 12), *Q74*- (*n* = 7)*,* and *Q23-DRD3*-HEK (*n* = 7) cells treated with PPX (6 h within 12 h after transfection). The densitometric analysis showed that PPX promotes a significant decrease in Q74 but not Q23 in *DRD3*-HEK cells, nor Q74 in HEK cells (Kruskal–Wallis followed by Dunn’s multiple comparison test). (**B**–**E**) Fluorescent labeling and quantitative analysis of the number of pyknotic nuclei (**B**,**D**) and immunofluorescent intensity of polyQ- and γH2AX expression in *Q74-DRD3*- and *Q23-DRD3*-HEK cells treated with PPX (**C**,**E**). PPX reduced the number of pyknotic nuclei (**B**(**iv**,**v**),**D**) and polyQ and γH2AX immunofluorescent intensity (**C**(**iv**,**v**),**E**) in *Q74-DRD3*-HEK cells without affecting *Q23-DRD3*-HEK cells. Statistical analyses were performed using ANOVA followed by Bonferroni’s (number of pyknotic nuclei and H2AXγ labeling intensity) and Brown–Forsythe tests, followed by Sidak’s (polyQ labeling intensity) multiple comparison tests. Arrows in (**B**) indicate pyknotic nuclei. A.U., arbitrary units. Bar: in (**B**(**v**)) (for (**B**(**i**–**v**))): 50 µm; in (**C**(**v**)) (for (**C**(**i**–**v**))): 10 µm. *n* = number of experimental repeats; *** *p* < 0.001. ns, not significant.

In line with these results, PPX (0.1 µM and 10 µM, 24 h) also significantly reduced Q74 levels in *Q74-DRD3*-HEK cells (Figure 5A, left). Still, no changes in Q74 were found in *Q74*-HEK cells (Figure 5A, middle) or Q23 levels in *Q23-DRD3*-HEK cells (Figure 5A, right), indicating that PPX effects depend on DRD3 and pathogenic polyQ expression. In addition, Q74 clearance was also activated in *DRD3*-HEK by another DRD2/DRD3 agonist, 7OH-DPAT (0.01 µM and 1 µM, 24 h; Figure S2A). Since polyQ can cause DNA damage and activate the DNA damage response pathway [45,46], we further investigated whether PPX protects against the genotoxic effects of Q74. DNA damage was assessed by counting the number of pyknotic nuclei resulting from chromatin condensation in DAPI-stained cells and DNA damage response by the expression of the phosphorylated (activated) form of the histone H2A variant H2AX at serine 139 (γH2AX), an early and sensitive marker of DNA repair [46]. As shown in Figure 5B–E, unlike *DRD3*-HEK cell cultures, where pyknotic nuclei and γH2AX immunoreactivity were virtually absent or negligible (Figure 5B(i),C(i)), some cells (~3%) showed pyknotic nuclei (Figure 5B(ii),D) and became γH2AX-immunoreactive (Figure 5C(ii),E) in *Q23-DRD3*-HEK cell cultures, suggesting that polyQ transfection by itself causes slight DNA damage. As expected, the number of pyknotic nuclei (13.7%) and the intensity of γH2AX immunolabeling in *Q74-DRD3*-HEK cells were significantly higher than in *Q23-DRD3*-HEK cells (Figure 5B(iv),C(iv)–E). In addition, consistent with the decline in Q74 expression, PPX also promoted a significant decrease in the number of pyknotic nuclei and the intensity of γH2AX labeling (Figure 5B(v),C(v)–E). PPX did not affect DNA damage or the DNA damage response induced by *Q23* transfection (Figure 5B(iii),C(iii)–E). These results indicate that prolonged DRD3-mediated autophagy promotes mHTT and Q74 clearance, protecting striatal MSNs and HEK cells from their cytotoxic effects, without affecting WT mice or *Q23-DRD3*-HEK cells.

### 3.3. DRD3-Induced Autophagy Involves Different Signaling Pathways in Healthy and polyQ-HTT-Challenged Cells

Previous studies have shown that DRD3-induced autophagy is mediated by mTORC1 inhibition under physiological conditions [20]. However, others also suggest that mTORC1 is constitutively overactivated in mHTT-expressing cells [21]. Confirming this idea, we found that mTORC1 was hyperphosphorylated in R6/1 mice and *Q74-DRD3*-HEK cells compared with WT mice and *Q23-DRD3*-HEK cells, respectively (Figure S2B,C). This condition may prevent mTOR from being efficiently dephosphorylated/inhibited by DRD2/DRD3 agonists, thereby inducing autophagy in experimental models of HD, suggesting that alternative signaling pathways may be activated. Since autophagy may also be initiated by AMP-activated protein kinase (AMPK) activation [47,48], and, like mTORC1, it is regulated by G protein-coupled receptors, including dopamine receptors [49,50,51], the effects of PPX were explored in both pathways. It is worth noting that AMPK maintains an opposing relationship with mTORC1 in regulating cellular metabolism and autophagy. Unlike mTORC1, AMPK is activated in response to nutrient depletion to restore energy resources, thereby inhibiting mTORC1 activity and cell growth and directly promoting autophagy [52,53]. The opposing effect of AMPK and mTORC1 on autophagy is associated with their selectivity for ULK1 phosphorylation sites. AMPK phosphorylates ULK1 at diverse residues, including Ser317, Ser555, and Ser777 and mTORC1 at Ser757, which prevents AMPK-ULK1 interaction and autophagy activation [53,54]. mTORC1 kinase activity was assessed by monitoring the phosphorylation of mTOR at Ser2488 [55], its downstream kinase p70S6K at Thr389, and its autophagy effector ULK1 at Ser757. AMPK signaling was assessed by monitoring the phosphorylation of its catalytic subunit (PRKAA/AMPKα) at Thr172 and ULK1 at Ser555.

**Figure 6:**
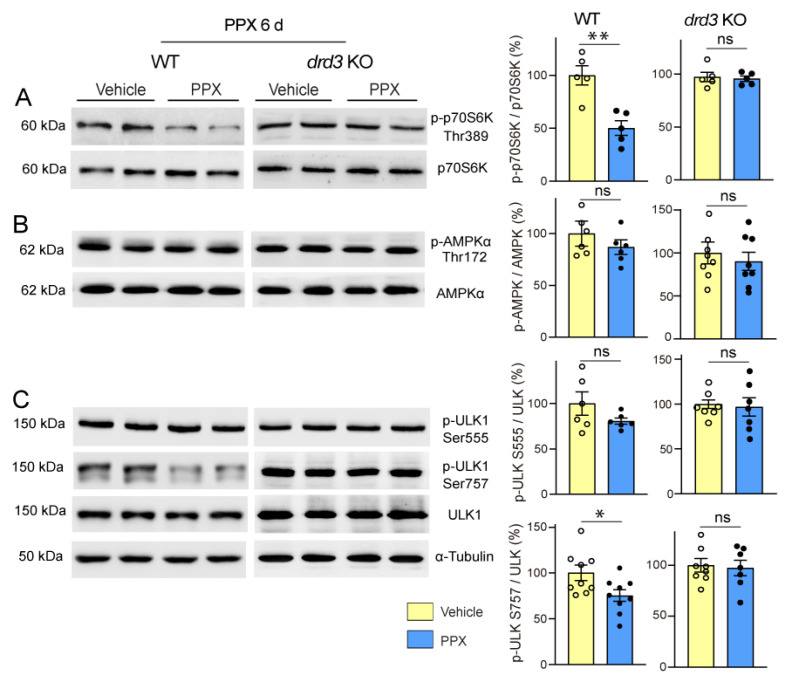
Short treatment with PPX (0.15 mg/kg, 6 days) induces mTORC1 inhibition through a DRD3-dependent mechanism. Western blot and densitometric analysis for the total and phosphorylated forms of p70S6K at Thr389 (**A**), AMPKα at Thr172 (**B**), and ULK1 at Ser at 757 and Ser555 (**C**) in striatal extracts of WT and *drd3*KO mice. PPX promoted a decrease in Thr389-p70S6K and Ser757-ULK phosphorylation in WT mice without affecting Thr172-AMPKα and Ser555-ULK phosphorylation. No phosphorylation changes were detected in *drd3*KO mice. *n* = 5–9 mice per experimental group, unpaired *t*-test; ns = non-significant; * *p* < 0.05; ** *p* < 0.01.

**Figure 7:**
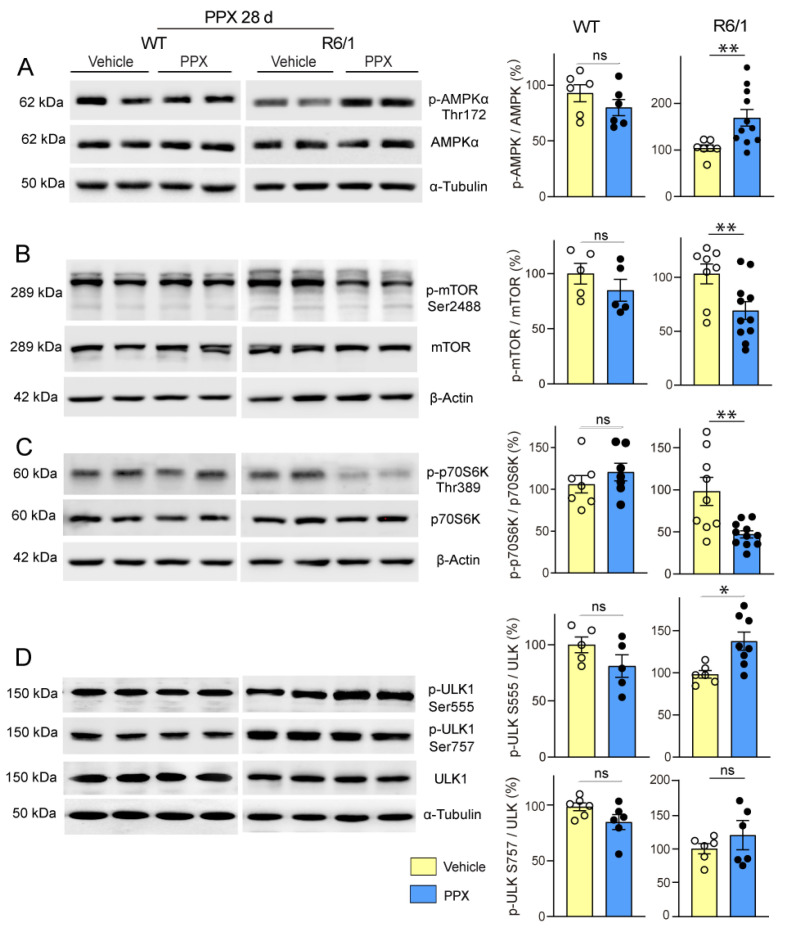
Prolonged treatment with PPX (0.15 mg/kg, 28 days) promotes AMPK activation and the partial inhibition of mTORC1 in the striatum of R6/1 mice but not in WT mice. Western blot and densitometric analysis for the total and phosphorylated forms of AMPKα at Thr172 (**A**), mTOR at Ser2488 (**B**), p70S6K at Thr389 (**C**), and ULK1 at Ser757 and Ser555 (**D**). PPX induces phosphorylation of Thr172-AMPKα (**A**) and Ser555-ULK1 (**D**), and dephosphorylation of Ser2488-mTOR (**B**) and Thr389-p70S6K (**C**) in the striatum of R6/1 mice without changes in Ser757-ULK1. No changes were detected in WT mice. *n* = 6–11 mice per experimental group; unpaired *t*-test. ns = non-significant; * *p* < 0.05; ** *p* < 0.01.

Firstly, we found that PPX induced dephosphorylation of the mTORC1 downstream kinase p70S6K at Thr389 (pThr389-p70S6K/p70S6K ratio) and its autophagy effector ULK1 at Ser757 (pSer757-ULK1/ULK1 ratio) in the striatum of WT mice without affecting Thr172-AMPKα and Ser555-ULK phosphorylation. No changes were detected in *drd3*KO mice (Figure 6). In addition, paralleling the temporary activation of autophagy markers in WT mice (see Figure 2C,D), the phosphorylation of mTOR and its downstream targets p70S6K and ULK1 at Ser757 returned to basal levels after 28 days of treatment (Figure 7, left; see also Figure S3). This indicates that under healthy conditions, DRD3-induced autophagy is transient and mTOR-dependent without significantly affecting AMPK signaling. Interestingly, both mTOR and AMPK pathways were altered by prolonged PPX treatment in R6/1. As shown in Figure 7, mTOR signaling was partially inhibited, as indicated by the dephosphorylation of mTOR and p70S6K at Ser2488 and Thr389, respectively, but not ULK1 at Ser757. In addition, unlike WT mice, the phosphorylation of both Thr172-AMPKα and Ser555-ULK1 was increased (Figure 7A,D), suggesting that in R6/1 mice, DRD3-induced autophagy is mediated by AMPK activation rather than mTOR inhibition.

**Figure 8:**
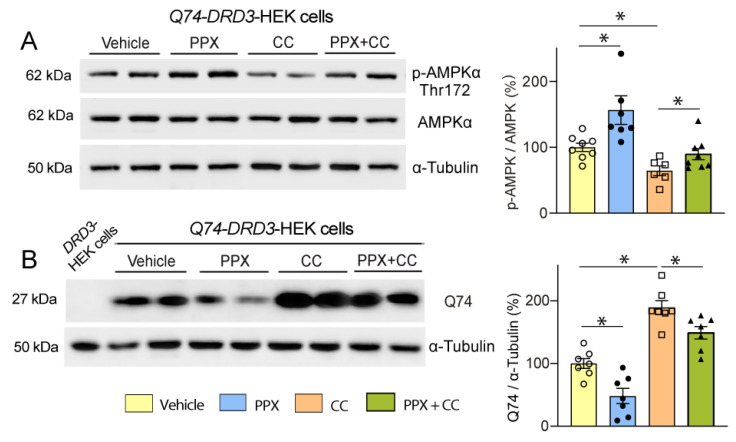
DRD3 promotes Q74 clearance through AMPK. Western blot and densitometric analysis of the total and phosphorylated forms of AMPKα at Thr172 (**A**) and Q74 in *Q74-DRD3*-HEK cells (**B**). PPX (0.1 µM, 6 h) induces Thr172-AMPKα phosphorylation ((**A**), lanes 3 and 4), rescues Thr172-AMPKα dephosphorylation induced by the AMPK inhibitor compound C (CC; 2 µM, 6 h; (**A**), lanes 7 and 8), and reduces Q74 levels in *Q74-DRD3*-HEK cells ((**B**), lanes 4 and 5) and *Q74-DRD3*-HEK cells treated with CC ((**B**), lanes 8 and 9). Lane 1 in (**B**), polyQ-untransfected *DRD3*-HEK cells. *n* = 6–8 experimental repeats. Statistical analyses were performed using ANOVA followed by the Fisher LSD test in the study of AMPKα phosphorylation and ANOVA followed by the Holm–Sidak multicomparison test in the study of Q74 levels. * *p* < 0.05. Analysis of S2488-mTOR was inconclusive since no differences among treatments resulted from either mTOR-independent AMPK activation or the recovery of transient mTOR inhibition in non-*Q74* transfected cells.

To test the relevance of AMPK in the autophagic clearance of polyQ-HTT induced by PPX, *Q74*-*DRD3*-HEK cells were treated with PPX alone or in combination with the AMPK inhibitor compound C (CC, 2 µM, 6 h). As expected, PPX induced an increase in Thr172-AMPKα phosphorylation and partially rescued AMPKα phosphorylation from the loss caused by CC (Figure 8A). Likewise, AMPK inhibition provoked a rise in Q74 levels (Figure 8B, compare lanes 2 and 3 with lanes 6 and 7) that was mitigated by PPX (Figure 8B, compare lanes 6 and 7 with lanes 8 and 9), indicating that AMPK mediates the autophagy clearance of polyQ-HTT induced by PPX.

**Figure 9:**
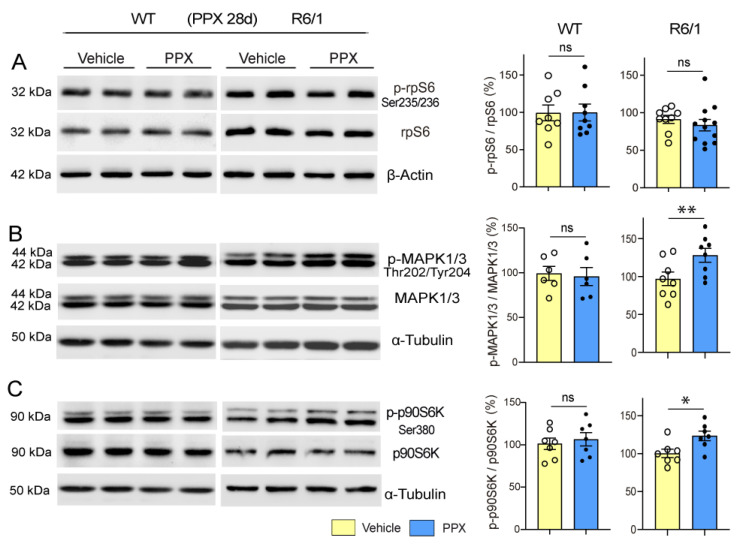
rpS6 activity is preserved and MAPK1/3 and p90S6K are activated in the striatum of R6/1 mice after prolonged treatment with PPX. Western blot for the total and phosphorylated forms of rpS6 at Ser235/236 (**A**), MAPK1/3 at Thr222/Tyr204 (**B**), and p90S6K at Ser380 (**C**) in WT and R6/1 mice. The densitometric analysis showed that Ser235/236-rpS6 phosphorylation was unaffected (**A**) and that PPX induced phosphorylations of Thr202/Tyr204-MAPK1/3 (**B**) and Ser380-p90S6K (**C**) in R6/1 mice. No changes were detected in WT mice. *n* = 6–12 mice per experimental group, unpaired *t*-test; ns = non-significant; * *p* < 0.05; ** *p* < 0.01.

Although AMPK mediates autophagy, mTORC1-p70S6K signaling remains inhibited in mHTT-expressing cells, which could be a worrying aspect of DRD3-induced autophagy given the pivotal role of this pathway in protein synthesis. However, despite mTORC1-p70S6K inhibition, the phosphorylation of Ser235/236-rpS6, the mTORC1-p70S6K target in the signaling cascade of protein synthesis, was not affected in R6/1 mice (Figure 9A), suggesting that alternative pathways may maintain rpS6 activity. Considering the crosstalk between mTORC1 and MAPK1/3 (mitogen-activated protein kinase) to preserve rpS6 activity [56,57], MAPK1/3-p90S6K signaling was also analyzed. As shown in Figure 9B,C, the phosphorylation of MAPK1/3 at Thr202/Tyr204 and p90S6K at Ser380 was increased, indicating that PPX also activates mTOR-MAPK1/3 crosstalk in the striatum of R6/1 mice.

## 4. Discussion

The results show that DRD3 activation can induce two forms of autophagy: a transient one in WT mice and *Q23-DRD3*-HEK cells and a persistent one in R6/1 mice and *Q74-DRD3*-HEK cells, which promotes the efficient clearance of mHTT/polyQ and protects striatal medium neurons and HEK cells from its cytotoxic effects without affecting healthy cells.

Unlike unselective autophagy, which sequesters and degrades bulky portions of the cytoplasm to maintain cell energy resources, selective autophagy aims to keep organelles functional and protect cells against toxic aggressions, including misfolded proteins. In selective autophagy, cargos are tagged by autophagy receptors that interact with the autophagosome marker LC3-II at the phagophore membrane, facilitating cargo sequestration and degradation [58,59]. p62 and TOLLIP are two receptors involved in several forms of selective autophagy [60,61] that recognize polyubiquitinated cargos via a UBA or CUE domain, respectively [41,58]. Although both receptors work cooperatively in the autophagic clearance of polyQ-proteins, TOLLIP is particularly involved and more efficient than p62 in removing ubiquitinated polyQs [40,41,62]. In parallel with LC3-II modulation, cargo receptors were reduced in both WT and R6/1 mice, indicating that selective autophagy was activated by PPX in both conditions, albeit with distinct temporal activation patterns. In WT mice, p62 and LC3-II were only transiently reduced, indicating a temporary reinforcement of basal autophagy not perpetuated by prolonged DRD3 stimulation. In R6/1 mice, p62 and TOLLIP were persistently reduced and LC3-II levels were persistently increased, indicating that in the presence of mHTT, DRD3-induced autophagy becomes a long-lasting phenomenon. The DRD3 dependence of PPX-induced autophagy and its prolongation only in cells expressing a pathogenic form of polyQ were corroborated in HEK cells. Autophagic flux was induced in *DRD3*- but not in untransfected or *DRD2*-transfected cells, and it became persistent in *DRD3*-cells expressing the pathogenic Q74-polyQ but not those expressing Q23-polyQ.

While p62 and LC3-II were reduced in WT mice, a sustained decrease in p62 and TOLLIP was paralleled by an increase in LC3-II in R6/1 mice. This suggests that under healthy conditions, both the flux and degradation of autophagosomes are transiently increased, but, in R6/1 mice, the rate of autophagosome synthesis exceeds that of degradation. Autophagosomes are synthesized at axon terminals and retrogradely transported to the soma, where they fuse with lysosomes to degrade their cargo [58,63]. Cargo recognition and autophagosome transport are regulated by endogenous HTT [64], which enhances their trafficking and degradation; in contrast, its mutated forms hinder this process [65,66,67]. Therefore, when autophagosome synthesis is enhanced in R6/1 mice, the response of autophagosome trafficking may be insufficient to meet the increased demand, resulting in autophagosome accumulation. A consequence of this deficit could be an increase in the size of intranuclear inclusions. While a large body of evidence supports the toxicity of soluble mHTT oligomers, the pathogenic significance of intracellular inclusions remains a matter of debate. The toxic effect of soluble mHTT oligomers comes from the complexity of their interactome. They can interact with proteins involved in critical cellular processes, including ribosome biogenesis, translation, transcription, and vesicle transport, interfering with these functions [68]. Regarding mHTT aggregates, studies from the first decade of the 2000s suggested a neuroprotective role, as the enlargement of inclusion bodies was associated with reduced soluble mHTT levels and improved proteasome function and cell survival [13,69]. More recent reports indicate that the protective or toxic potential of intracellular aggregates depends on their conformation and protein composition and that the composition of intranuclear inclusions differs from that of cytoplasmic inclusions [70]. Thus, although intranuclear inclusions maintain a fibrillary conformation that can induce the deformities of nuclear membranes, unlike cytoplasmic inclusions, they do not contain membranous structures of disrupted organelles, such as the endoplasmic reticulum and mitochondria [71]. Interestingly, these studies also show that the progressive increase in the size of intranuclear inclusions is not associated with an increase in toxicity or cell death, suggesting a lack of correlation between intranuclear inclusion size and toxicity. In line with these data, the enlargement of intranuclear inclusions in R6/1 mice did not hinder the achievement of a substantial decline in soluble mHTT and the recovery of DARPP-32 striatal expression and motor behavior. These findings, together with the decrease in Q74-polyQ and the consequent reversal of its genotoxic damage in HEK cells, suggest that DRD3 activation may be an effective way to induce autophagic clearance of mHTT/polyQ.

**Figure 10:**
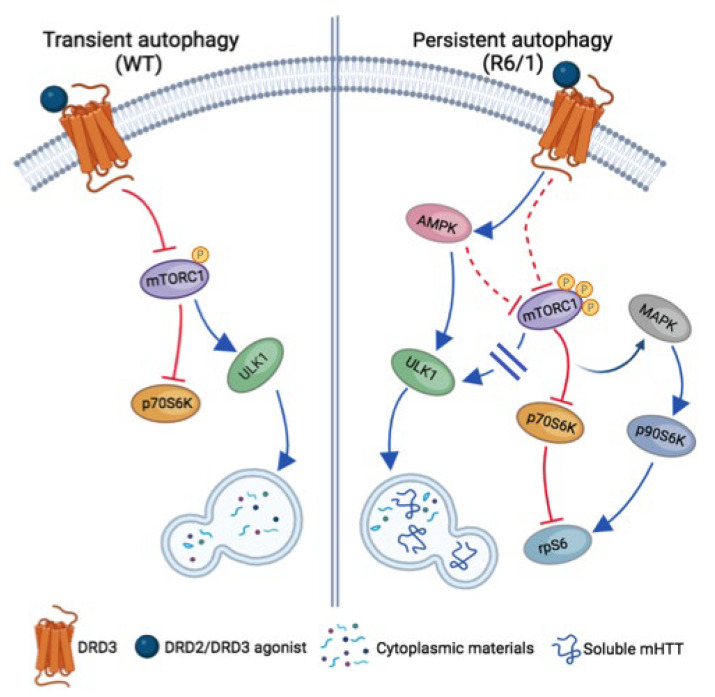
Schematic view of signaling pathways involved in transient and persistent DRD3-medited autophagy in WT and R6/1 mice, respectively. In WT mice (**left**), autophagy is transiently induced by mTORC1 inhibition with the subsequent inhibition of p70S6K and the activation of ULK1. In R6/1 mice (**right**), where mTORC1 is hyperphosphorylated, autophagy is persistently activated through AMPK with the direct activation of ULK1, promoting an effective clearance of soluble mHTT. In persistent autophagy, the mTORC1-p70S6K branch of mTORC1 signaling is also inhibited, but mTORC1-ULK1 signaling is not activated. In addition, the crosstalk between mTORC1-p70S6K and MAPK-p90S6K pathways is activated and rpS6 activity is preserved. Blue arrows and blocked red lines indicate the activation or inhibition of downstream targets, respectively. Dashed red lines indicate putative pathways of mTORC1 inhibition in persistent autophagy.

A further difference between DRD3-induced autophagy in WT and R6/1 mice is the signaling pathway underlying its activation (Figure 10). In WT mice, autophagy was coupled with transient mTORC1 inhibition, as indicated by the dephosphorylation of Thr389-p70S6K and Ser757-ULK1 after 6 days but not after 28 days of treatment. Previous cell studies indicate that classical autophagy, induced by starvation and rapamycin treatment, is also transient and mediated by mTORC1 inhibition, with subsequent mTORC1 reactivation, signaling autophagy termination, and protein synthesis recovery [72,73]. So, we can say that under non-pathological conditions, treatment with DRD2/DRD3 agonists promotes a form of transient classical/mTORC1-mediated autophagy. In R6/1 mice, where mTORC1 is constitutively hyperphosphorylated, PPX also provoked a persistent inhibition of mTORC1-p70S6K signaling, as indicated by the dephosphorylation of their Ser2488 and Thr389 residues, respectively. Still, the treatment did not affect the phosphorylation of its autophagic target Ser757-ULK1. In contrast, AMPKα, the catalytic subunit of AMPK, and ULK1 were phosphorylated at Thr172 and Ser555, respectively. Therefore, although the p70S6K limb of mTORC1 was inhibited, autophagy was maintained by the direct activation of AMPK-ULK1 signaling rather than by mTORC1 inhibition.

The meaning of AMPK in neurodegenerative diseases has long been controversial. Whereas some studies in experimental models of Parkinson’s and Alzheimer’s disease suggest that it can contribute to dopaminergic degeneration and Tau phosphorylation [74,75], others advocate neuroprotection through different mechanisms [76,77]. In the case of HD, AMPK activation has also been associated with either a higher vulnerability of striatal cells [78,79] or a decrease in soluble mHTT, an improvement in motor behavior, and an increase in survival time in R6/2 mice [80,81]. These discrepancies may be due to differences in the experimental approach and possible AMPK-independent effects of compounds used as activators [82,83]. Our experiments in mice indicate that AMPK activation is a downstream signaling effect of DRD3 associated with mHTT clearance and neuroprotection. Furthermore, PPX induced Thr172-AMPKα phosphorylation and a reduction in Q74-polyQ, as well as its toxic effects, in HEK cells, whereas AMPK inhibition led to Q74-polyQ accumulation. Therefore, we can conclude that AMPK plays a central role in the DRD3-induced autophagic clearance of mHTT/polyQ.

The coexistence of AMPK activation and mTORC1 inhibition suggests that PPX promotes crosstalk between both signaling pathways in the striatum of R6/1 mice. AMPK-mTORC1 crosstalk is usually activated in healthy cells in response to nutrient demands. Both pathways maintain a dynamic interplay, activating either protein synthesis or autophagy through negative feedback at different levels of their signaling cascade. For instance, ULK1 can inhibit mTORC1 activity by dephosphorylating Thr389-p70S6K [84,85]. Additionally, ULK1, p70S6K, and mTORC1 can also inhibit AMPK through inhibitory phosphorylations in their respective subunits [86,87]. Our results indicate that crosstalk between AMPK and mTORC1 is also activated and maintained for an extended period in mHTT-challenged cells by engaging a G protein-coupled receptor. The phosphorylation pattern suggests that AMPK may inhibit mTORC1-p70S6K while simultaneously activating ULK1. However, given the complexity of their possible interactions, elucidation of the underlying mechanisms requires further study.

Since the mTORC1-p70S6K pathway regulates essential functions in the adult brain, including neurotransmission, synaptic plasticity, cognition, and stress response [88], its prolonged inhibition may have detrimental consequences for brain functions, counteracting the beneficial effects of the autophagic clearance of misfolded proteins. Interestingly, mTORC1-p70S6K inhibition was accompanied by further crosstalk between mTORC1 and MAPK, leading to the activation of p90S6K and the preservation of rpS6 activity. mTORC1-MAPK cross-inhibition was initially described in healthy and cancer cells treated with rapamycin [89]. Both pathways interact through positive and negative feedback to control cell metabolism and survival in response to different cues [90]. The activation of p90S6K resulting from mTORC1-p70S6K inhibition maintains rpS6 activity and protein synthesis, with the subsequent loss of antiproliferative effects of rapamycin if MAPK-p90S6K signaling is not simultaneously inhibited [91]. The evidence of this crosstalk means that, despite mTORC1 inhibition, protein synthesis is preserved in the striatum of R6/1 mice, contributing to the neuroprotective effect of DRD3-induced autophagy.

Given the central role of mHTT in the pathogenesis of HD, therapeutic approaches are focused on reducing mHTT levels in the brain of HD patients. Prompted by striking results in preclinical studies, gene therapy is considered the most promising strategy. Several clinical trials have been initiated in the last decade, mainly using antisense oligonucleotides and microRNAs. Some data allow us to be optimistic about their future. Still, most of these trials have been terminated due to the lack of therapeutic efficacy and the emergence of relevant side effects, highlighting the need to investigate alternative treatments. Our results suggest that DRD2/DRD3 agonists can be an effective disease-modifying therapy in HD, activating selective autophagy through DRD3. The fact that when acting on a specific neuronal population, healthy cells are only transiently affected, and that polyQ-challenged cells activate compensatory crosstalk to maintain rpS6 activity, minimizes potential risks of prolonged autophagy, suggesting that DRD3-induced autophagy is an efficient and safe way of removing mHTT from striatal neurons. DRD2/DRD3 agonists are currently used in clinical neurology as symptomatic treatment for neurological conditions such as Parkinson’s disease and restless leg syndrome [25]. Their pharmacokinetics, dosage, side effects, and brain concentration after oral administration are well known. These data can simplify the design of clinical trials, reducing the costs of verifying the benefit of these compounds in HD patients.

## 5. Conclusions

Our studies in cell and animal models of HD show that DRD2/DRD3 agonists promote two forms of autophagy through a DRD3-dependent mechanism. One, in WT mice and healthy cells, is transiently activated via mTORC1 inhibition, resembling classical autophagy induced by starvation and rapamycin. The other, in R6/1 mice and polyQ-expressing cells, is persistent and mediated by direct AMPK-ULK1 activation, also involving regulatory cross-talks between AMPK, mTORC1, and MAPK with the preservation of rpS6 activity. Furthermore, DRD3-induced autophagy promotes a substantial decrease in soluble mHTT/PolyQ, thereby protecting striatal neurons and HEK cells from its cytotoxic effect, as indicated by the recovery of striatal DARPP-32 expression and motor behavior in mice and the reversal of genotoxic damage in HEK cells. Bearing in mind the central role of striatal MSN degeneration in HD, these findings suggest that DRD3-induced autophagy may have disease-modifying potential in HD.

## Data Availability

Raw data associated with this study, including uncropped Western blots, are available from the University of La Laguna (https://data.mendeley.com/datasets/x26v2kk2bk/3 (accessed on 10 April 2025)). Further details about procedures are available from the corresponding author upon request.

## Supplementary Materials

The following supporting information can be downloaded at https://www.mdpi.com/article/10.3390/cells14090652/s1: Figure S1: Stable expression of *DRD2* and *DRD3* and transient expression of *GFP-Q23* and *HA-Q74* in HEK cells; Figure S2: Effects of 7OH-DPAT on Q74 expression (A) and mTOR hyperphosphorylation in R6/1 mice (B) and Q74-expressing cells; Figure S3. Summary of changes in kinase phosphorylation and autophagy markers in mice and transfected HEK cells

## Abbreviations

AMPK, AMP-activated protein kinase; CQ, chloroquine; DARPP-32, dopamine- and cAMP-regulated phosphoprotein; DRD2, dopamine receptor D2; DRD3, dopamine receptor D3; EGFP, enhanced green fluorescent protein; GFP, green fluorescent protein; HD, Huntington’s disease; HEK, human embryonic kidney; MAP1LC3/LC3, microtubule-associated protein 1 light chain 3; MAPK1/3, mitogen-activated protein kinase 1/3; mHTT, mutated huntingtin; MSNs, medium spiny neurons; mTOR, mechanistic target of rapamycin kinase; mTORC1, mTOR complex 1; PPX, pramipexole; PRKAA/AMPKα, α (catalytic) subunit of AMPK; rpS6, ribosomal protein S6; RPS6KA/p90S6K, ribosomal protein S6 kinase A; RPS6KB/p70S6K, ribosomal protein S6 kinase B1; SQSTM1/p62, sequestosome 1; TOLLIP, Toll-interacting protein; ULK1, unc-51 like autophagy activating kinase 1; WT, wild type; γH2AX: phosphorylated H2AX at Ser139.
