# Supplementary material for: Dopamine Receptor D3 Induces Transient, mTORC1-Dependent Autophagy That Becomes Persistent, AMPK-Mediated, and Neuroprotective in Experimental Models of Huntington’s Disease
Source: Cells. 2025 Apr 29;14(9):652. doi: 10.3390/cells14090652 (PMC12071662; doi:10.3390/cells14090652)
Supplement: Supplementary file 1 [file cells-14-00652-s001.zip › cells-3564269-supplementary.pdf]

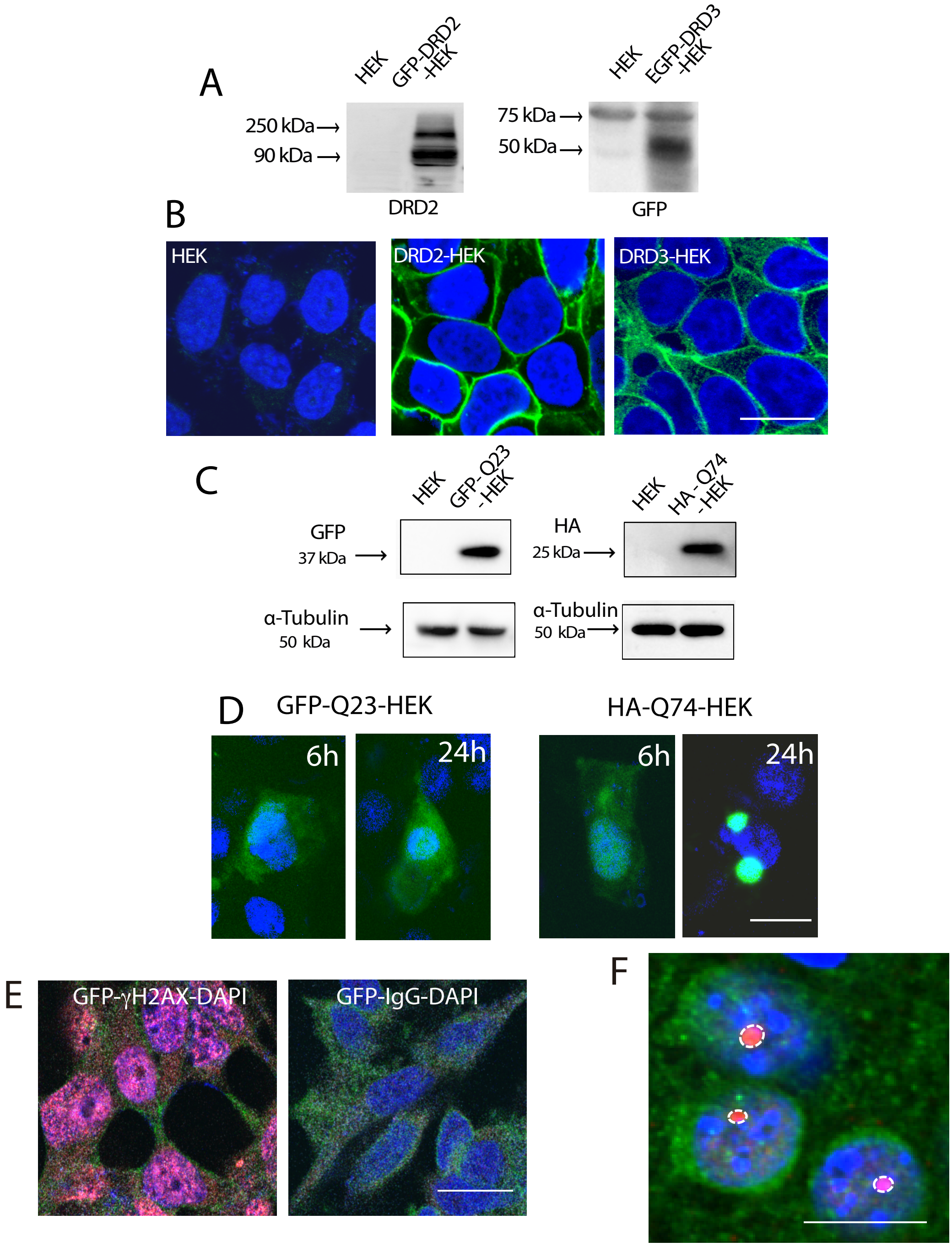

Figure S1. (**A, B**) Stable expression of DRD2 and DRD3 in HEK cells. Western-blot for DRD2 and GFP (**A**) and fluorescence for GFP (**B**) in untransfected (left), GFP-DRD2- (middle) and EGFP-DRD3- (right) transfected HEK cells. (**C, D**) Transient expression of GFP-Q23 and HA-Q74 in HEK cells. (**C**) Western blot for GFP and HA. (**D**) Q23 and Q74 expression 6 hours and 24 hours after transfection. Unlike Q23, Q74 is aggregated after 24 in most transfected cells. (**E**) Positive (left) and negative (right) control experiments for γH2AX immunofluorescent labelling in DRD3-HEK cells treated with doxorrubicin. The primary antibody was substituted by preimmune IgG (IgG) in negative controls. (**F**) Double labeling for DARPP32 (green) and HTT using EM48 antiserum (red) in MSNs. Dotted lines indicate intranuclear inclusions as encircled for the quantitative analysis of their size. Nuclei were stained with DAPI (blue).
Bar in B, D, E, and F, 10 µm.

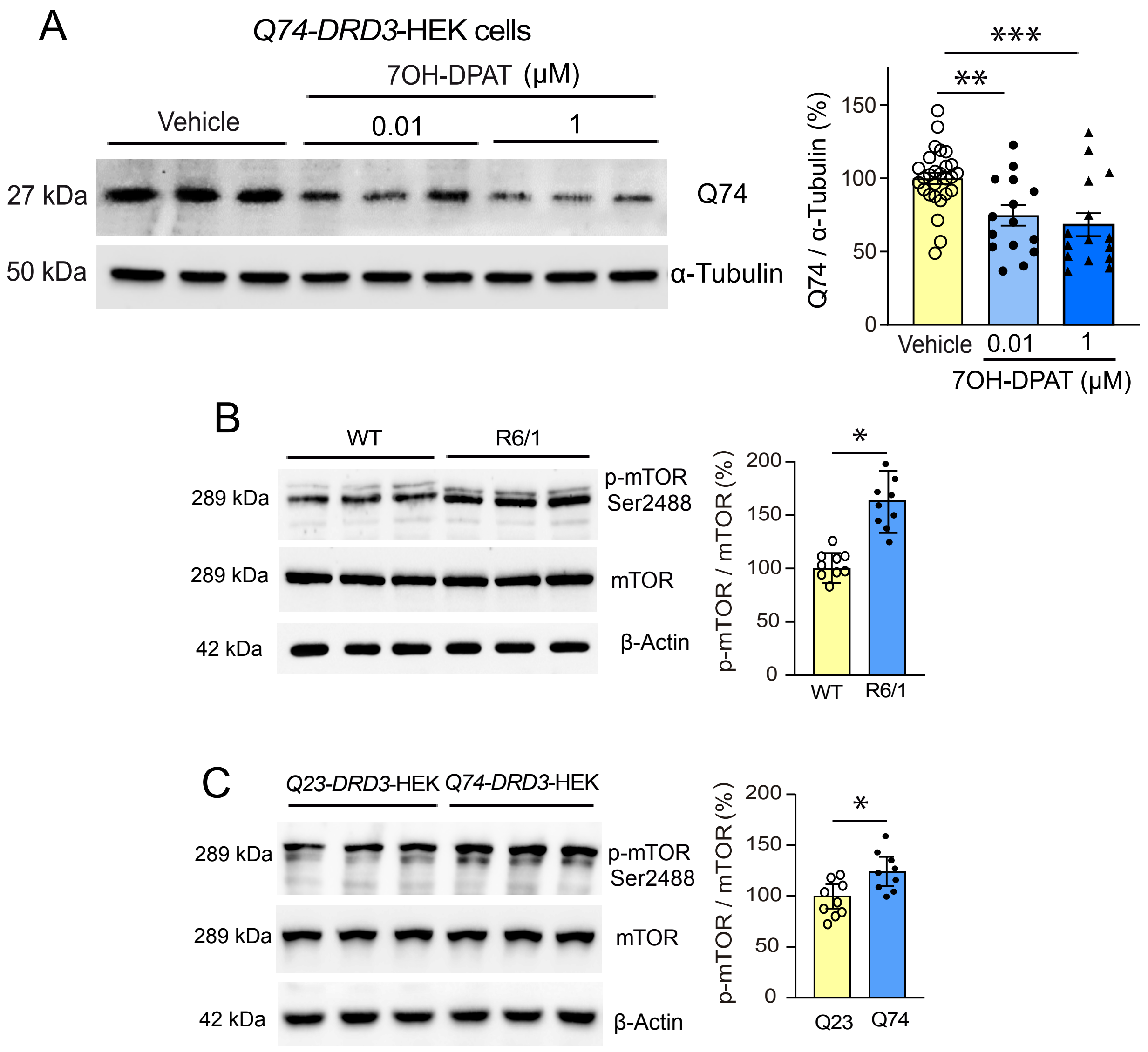

Figure S2. (**A**) 7OH-DPAT promotes Q74 clearance in DRD3-HEK cells. Western blot for polyQ in Q74-DRD3-HEK cells treated with 0.01µM and 1µM 7OH-DPAT. The densitometric analysis shows that 7OH-DPAT promotes a significant decrease of Q74. ANOVA followed by Tukey's multiple comparison test. n = 15-19 experimental repeats. (**B**, **C**) mTOR is hyperphosphorylated in R6/1 mice and Q74-DRD3-HEK cells. Western-blot for mTOR and p-mTOR en WT and R6/1 mice (**B**, Mann-Whitney test, n= 9), and Q23- and Q74-DRD3-HEK cells, (**C**, *t*-test, n=9). * $p < 0.05$; ** $p < 0.01$; *** $p < 0.001$.

KINASE PHOSPHORYLATION
(PPX vs. vehicle)

	S2488-mTOR	T389-p70S6K	T172-AMPK	Ser757-ULK1	Ser555-ULK1	T202/Y204 MAPK1/3	S380-p90S6K
**WT 6 days**		↓	=	↓	=		
**drd3 KO 6 days**		=	=	=	=		
**WT 28 days**	=	=	=	=	=	=	=
**R6/1 28 days**	↓	↓	↑	=	↑	↑	↑

AUTOPHAGY MARKERS
(PPX vs. vehicle)

	LC3-II	p62	TOLLIP
**WT 6 days**	↓	↓	=
**drd3 KO 6 days**	=	=	
**WT 28 days**	=	=	=
**R6/1 6 days**	↑	=	↓
**R6/1 28 days**	↑	↓	↓

AUTOPHAGY MARKERS
(PPX+CQ vs. CQ)

	**HEK (4h)**	***DRD2*-HEK (4h)**	***DRD3*-HEK (4h)**	***Q23-DRD3*-HEK (4h)**	***Q74-DRD3*-HEK (4h)**	***DRD3*-HEK (24h)**	***Q23-DRD3*-HEK (24h)**	***Q74-DRD3*-HEK (24h)**
**LC3-II**	=	=	↑	↑	↑	=	=	↑

Figure S3. Summary of changes in kinase phosphorylation and autophagy markers in mice and HEK293 cell lines with ectopic expression of dopamine receptors and polyQ proteins.
